# Encouraged

**Published:** 1893-06

**Authors:** 


### ENCOURAGED.

We present our grateful acknowledgments to our esteemed exchange, the *Medical and Surgical Reporter* of Philadelphia, for the kind words contained in its issue of May 20. Under the caption, "Medical Legislation in Georgia," our good contemporary says:

"THE ATLANTA MEDICAL AND SURGICAL JOURNAL has exercised a powerful influence towards securing a higher medical standard in Georgia. The current number contains a paper recently read by Dr. Grandy before the Georgia Medical Association presenting the necessity for an Examining Board. The bill for the establishment of a Board of Medical Examiners in Georgia, after encountering rabid opposition from sources common all over the United States, passed the Senate by nearly four-fifths majority but got tied up in the Committee of the House so that further action was postponed until the next meeting of the legislature. This ought to be encouraging to the gentlemen interested. They commenced operations last November and it would be miraculous to enlighten an ordinary legislature on this subject in so short a period. Had the present set-back been absolute it would only have delayed victory, for these advocates of progress could not accept defeat. To give up is not characteristic of the species."

In reproducing this paragraph we have no further object than to encourage those who are working and who will work for the desired legislation. We especially commend it to the Committee on Legislation appointed by the Association. This committee consists of the following gentlemen :

Third District—Dr. R. O. Engram, Chairman, Montezuma.
First District—Dr. R. J. Nunn, Savannah.
Second District—Dr. T. M. McIntosh, Thomasville.
Fourth District—Dr. W. L. Bullard, Columbus.
Fifth District—Dr. J. McFadden Gaston, Atlanta.
Sixth District—Dr. Mark H. O'Daniel, Macon.
Seventh District—Dr. J. B. S. Holmes, Rome.
Eighth District—Dr. S. C. Benedict, Athens.
Ninth District—Dr. W. B. Tate, Tate.
Tenth District—Dr. E. C. Goodrich, Augusta.
Eleventh District—Dr. J. A. Dunwoody, Brunswick.

We believe that these gentlemen will give their best energies to the success of this movement. With proper and earnest effort there can be little doubt as to the result. When this matter is presented squarely to the attention of the legislature we are sure that it cannot fail to appreciate the necessities of the situation, the opposition of certain parties to the contrary notwithstanding. A Board of Medical Examiners is not a matter of expediency; it is a *necessary public policy.*

